# Change in Finances, Peer Access, and Mental Health Among Trans and Nonbinary People During the COVID-19 Pandemic

**DOI:** 10.1089/lgbt.2022.0296

**Published:** 2023-12-04

**Authors:** Monica A. Ghabrial, Ayden I. Scheim, Caiden Chih, Heather Santos, Noah James Adams, Greta R. Bauer

**Affiliations:** ^1^ Epidemiology and Biostatistics, Schulich School of Medicine & Dentistry, Western University, London, Ontario, Canada.; ^2^ Department of Epidemiology and Biostatistics, Dornsife School of Public Health, Drexel University, Philadelphia, Pennsylvania, USA.; ^3^ Mechanical and Industrial Engineering and University of Toronto, Toronto, Ontario, Canada.; ^4^ Ontario Institute for Studies in Education, University of Toronto, Toronto, Ontario, Canada.; ^5^ Center for Applied Transgender Studies, Chicago, Illinois, USA.; ^6^ Transgender Professional Association for Transgender Health, Canada.; ^7^ Institute for Sexual and Gender Health, University of Minnesota Medical School, Minneapolis, Minnesota, USA.

**Keywords:** community access, COVID-19 pandemic, poverty and financial loss, trans and nonbinary (TNB) mental health

## Abstract

**Purpose:**

Due to structural transphobia, trans and nonbinary (TNB) individuals were particularly vulnerable to the negative effects of social isolation and financial instability resulting from COVID-19. The present study examined the effect of change in finances and access to TNB peer gatherings on anxiety and depression during the COVID-19 pandemic.

**Methods:**

Participants were 18 years and older (mean = 30) and completed prepandemic baseline (Fall 2019) and pandemic follow-up (Fall 2020) surveys. Multivariable regressions examined associations between mental health and change in (1) finances and (2) access to TNB peer gatherings (in person or online).

**Results:**

Of 780 participants, 50% reported that the COVID-19 pandemic had a negative impact on personal income and 58.3% reported negative impact on access to TNB peer gatherings. Depression and anxiety symptoms increased from prepandemic to follow-up, and most participants were above measurement cutoffs for clinical levels at both time points. Change in finances and access to TNB peer gatherings interacted with prepandemic depression scores to predict depression symptoms during the COVID-19 pandemic. For participants with high prepandemic depression scores, financial stability predicted pandemic depression scores comparable to that predicted by negative financial change. No interaction was found between these variables when predicting anxiety symptoms during the COVID-19 pandemic.

**Conclusion:**

Findings underscore the influence of inequality and prepandemic mental health when considering the impact of COVID-19 on wellbeing. Results suggest need for multifaceted programs and services, including financial support and meaningful TNB community engagement, to address barriers to health equity posed by systematic gender oppression.

## Introduction

Trans and nonbinary (TNB) people face discrimination and systemic oppressions that can lead to increased victimization and reduced economic stability, health care access, education achievement, access to community resources, and availability of social support.^1–7^ The onset of the COVID-19 pandemic brought about abrupt closures, job loss, social distancing measures, and other interruptions to daily life,^8^ which increased social isolation, financial insecurity, and stress at a population level.^9,10^ The COVID-19 pandemic magnified the vulnerability of historically excluded populations to the negative effects of a global crisis. Structural transphobia may have positioned TNB communities to be more severely affected by changes in social and financial security and mental health.^11^

Due to gender discrimination and antitrans stigma, TNB people are denied employment, refused promotions, and face workplace harassment.^5,12,13^ They similarly face housing insecurity, through refusal to rent from landlords, displacement from unsupportive family homes, and exclusion from and lack of safety within shelter systems.^3,14^ When the COVID-19 pandemic began, being more likely to work in service jobs (e.g., food service, retail) further exposed TNB people to health risks and job precarity.^15,16^ These structural vulnerabilities also increase financial difficulties with regard to affording gender-affirming surgeries, medications, and supplies.^17^ Barred access to this care can, in turn, have a direct impact on mental health.^18–20^

Social support may buffer the minority stressors encountered by TNB people.^21^ TNB individuals with low social support have the highest likelihood of discrimination-associated suicidal ideation compared with those with moderate or high support.^21^ Results from a systematic review on social support experienced by TNB people demonstrate that peer support is the source most commonly associated with better health and wellbeing.^22^ However, the disruption of day-to-day activities caused by COVID-19 restricted access to community and social gatherings,^11,20^ while some TNB people were forced to lockdown with unsupportive family.^15,23^ Compared with cisgender youth, TNB youth report more mental health and substance use service disruptions along with less social support amidst the pandemic.^11^

Existing COVID-19 research suggests that mental health was dramatically affected during the pandemic and that prepandemic mental health is a meaningful predictor of pandemic mental health.^24^ There is, however, limited information on the effects of this global crisis within a context of structural transphobia, and how financial and community loss may have impacted mental health in this structurally excluded population already entering the pandemic with disproportionate levels of depression and anxiety.^25,26^ Using longitudinal data, we investigated the effect of self-reported changes in finances and access to TNB peer gatherings (online or in-person) on depression and anxiety symptoms among TNB individuals during COVID-19, and examined if this relationship was moderated by prepandemic mental health symptoms.

## Methods

### Sample and procedure

Trans PULSE Canada (TPC) was a community-based, national study on wellbeing among TNB people, developed through intensive consultation process by nine priority population teams (TNB people who are Indigenous, nonbinary, people of Color, sex workers, older adults, youth, rurally living, disabled, or immigrants, refugees, and newcomers).^27^ Prepandemic data were collected from September to November 2019 from 2873 Canadian residents who were recruited using a multimode convenience sampling approach. The survey was promoted on social media, listservs, at community agencies and events, and through Peer Research Associate outreach. Participants completed either the full (∼60 minutes) or short-form (∼10 minutes) survey, which included only key items from each section of the full survey. After the baseline survey, 1190 participants consented to be recontacted to participate in additional research projects. In October 2020, 820 participants who had agreed to be recontacted completed a follow-up on the impact of the COVID-19 pandemic, for which they received a $20 gift card.

TPC was approved by the Research Ethics Boards of Western University, Unity Health Toronto, Wilfrid Laurier University, and the University of Victoria. The COVID-19 follow-up was approved at Western University, Wilfrid Laurier University, University of Victoria, Universal Health Network, University of British Columbia, and Drexel University. Participants provided informed consent. This article uses data from individuals who completed the prepandemic survey and the COVID-19 follow-up (*n* = 780). Participants included in analyses were 18 years or older at baseline, due to our focus on financial change during the COVID-19 pandemic, with the postulation that youth under 18 would be more likely to be financially dependent on a guardian(s), and thus less likely to experience personal financial loss.

### Measures

#### Sociodemographic variables

Participants responded to sociodemographic characteristic questions, including age, sex assigned at birth, immigration history, province, education, employment, and living situation. Respondents reporting that they identify as a person of Color (PoC) or that they are perceived or treated as a PoC in Canada were coded as PoC, and those who did not were coded as non-PoC. Individuals who reported being First Nations, Inuit, Métis, or otherwise Indigenous in Canada were coded as Indigenous, and all others were coded as non-Indigenous. Participants were asked to select a gender identity from the following options: (1) man or boy, (2) woman or girl, (3) Indigenous or other cultural gender identity (e.g., two-spirit), (4) nonbinary, genderqueer, agender, or a similar identity.

#### Income and needs

Participants reported income using categories that were provided in increments ranging from $5000 to $50,000 (e.g., <$10,000, $10,000 to <$15,000, $60,000 to <$80,000, $100,000 to <$150,000, $150,000 or over). We estimated numerical income for each participant by assigning the midpoint of their selected income category (e.g., for the category $20,000 to <$30,000, participants were assigned the amount of $25,000). For participants who selected the lowest or highest income category (<$10,000; $150,000 or more), they were assigned $9999 and $150,000, respectively. Income-to-needs ratio was calculated by dividing this integer by the reported number of individuals supported by the income, including the participant. To determine low-income category (Yes/No), we compared income and number supported to the Statistics Canada low-income measure.^28^

### Mental health

#### Depression

Prepandemic and pandemic depression symptoms were assessed using the 10-item Center for Epidemiologic Studies Depression Scale (CESD-10).^29^ Items are rated in frequency during the past week on a scale of 0 (*rarely or none of the time*; *less than 1 day*) to 3 (*most or all of the time*; *5–7 days*). Final scores are calculated by reverse-scoring as needed and adding item ratings (range = 0–30). A final score of 10 or higher is generally used as a cutoff for significant depression symptoms.^29^ Internal consistency was high at prepandemic baseline (Cronbach's alpha, *α* = 0.86) and at pandemic follow-up (*α* = 0.86).

#### Anxiety

Prepandemic and pandemic anxiety were assessed using the five-item Overall Anxiety Severity and Impairment Scale (OASIS), which inquires after anxiety symptoms and the impact of anxiety on daily life over the past week.^30^ Items are ranked in severity on a 5-point scale (0 = *Not at all*, 4 = *Extreme*) and summed (range = 0–20). A total score of 8 or higher is generally used as a cutoff for significant anxiety symptoms.^30^ Internal consistency was high in the present sample at prepandemic baseline (*α* = 0.89) and pandemic follow-up (*α* = 0.88).

#### Social support and living situation

We assessed social support using the eight-item modified Medical Outcomes Study Social Support Scale (mMOS-SS).^31^ Items are rated on a five-point scale (*Never* to *Constantly*). The mMOS-SS has two subscales, Tangible and Emotional support, which can be considered separately or as a whole. We averaged all eight items for an overall score, with higher scores indicating more social support. The mMOS-SS had high internal consistency at prepandemic baseline (*α* = 0.91). Participants were also asked if conditions related to COVID-19 had caused them to live with a household member who is unsupportive of their gender (1 = *Yes*, 2 = *No*).

#### Change in finances due to the COVID-19 pandemic

On the COVID-19 follow-up, participants were asked two questions about financial change, which were adopted from Statistics Canada surveys.^32^ The first of these items asked participants to rank the impact that COVID-19 had on their ability to meet financial obligations or essential needs. The second item asked participants to rank the impact that COVID-19 had on personal income from employment. Both items were ranked on an 8-point scale (*major negative impact, moderate negative impact, minor negative impact, no impact, minor positive impact, moderate positive impact, major positive impact, too soon to tell*). *Too soon to tell* was recoded as a missing value. To create a composite item for financial change during COVID-19, we combined these two items using the following guidelines.

If participants reported a major or moderate negative impact on both items, they were categorized as *negative change*. If participants reported neutral or minor positive impact on one item and moderate or major negative impact on another, they were categorized as *negative change.* If participants reported moderate or major positive impact on both items, they were categorized as *positive change*. If participants reported neutral or minor negative impact on one item and moderate or major positive impact on another, they were categorized as *positive change*.

If participants reported positive or negative minor impact or neutral on both, they were categorized as *no change*. If participants reported moderate or major positive on one item and moderate or major negative impact on the other, they received a missing score, as we did not have enough information to explain this discrepancy. If participants reported minor or neutral impact on one item and did not respond to the second item, they received a missing score.

#### Access to TNB peer gatherings

In the COVID-19 pandemic follow-up survey, we asked participants to rank how their access to TNB peer or friend gatherings (online or in person) had changed since March 12, 2020. This question was rated on a 3-point scale (*decreased, stayed the same, increased*).

### Analysis

#### Missing data

The percentage of missing values in relevant variables ranged from 0% to 6% of cases and relevant data were complete for 84.6% of participants. The pattern was nonmonotone, suggesting that missing data were not due to issues related to participant attrition during survey completion. For multivariable regression analyses, we conducted multiple imputation using fully conditional specification in SAS 9.4, producing 20 imputations. Outcome variables (pandemic depression and anxiety) were not imputed. Sensitivity analyses revealed similar outcomes when analyses were run with the Missing *Not At Random* command. Similar results were obtained when analyses were restricted to complete case data. Descriptive data (Table 1) were obtained before imputation.

**Table 1.** Participant Characteristics, Stratified by Change in Finances and Access to Trans and Nonbinary Peer Gatherings (*n* = 780)

	Total sample		Change in finances				Change in access to trans and nonbinary peer gatherings			
	*n*	Weighted % (95% CI)	Negative (95% CI)	No change (95% CI)	Positive (95% CI)	*χ* ^2^	Decrease (95% CI)	No change (95% CI)	Increase (95% CI)	*χ* ^2^
Age						0.2129				0.9638
18–20	39	5.67 (3.86–7.47)	53.68 (36.94–70.41)	39.81 (23.37–56.25)	6.52 (0–13.84)		63.22 (47.61–78.84)	29.57 (14.79–44.35)	7.21 (0–15.15)	
21–24	131	18.84 (15.75–21.94)	50.95 (41.48–60.42)	40.89 (31.55–50.24)	8.16 (3.07–13.25)		60.39 (51.34–69.45)	33.39 (25.12–42.75)	5.67 (1.66–9.68)	
25–34	307	40.81 (37.05–44.57)	42.20 (36.13–48.28)	49.84 (43.62–56.06)	7.96 (4.32–11.60)		56.52 (50.53–62.51)	33.81 (28.06–39.55)	9.67 (6.06–13.28)	
35–44	176	19.67 (16.75–22.59)	34.56 (26.50–42.630	57.49 (49.08–65.90)	7.94 (2.83–13.06)		58.43 (50.35–66.52)	33.63 (25.96–41.30)	7.94 (3.08–12.79)	
45–59	94	11.30 (8.94–13.67)	39.53 (28.77–50.28)	53.56 (42.56–64.57)	6.91 (1.66–12.16)		55.34 (44.42–66.26)	37.65 (27.38–47.92)	7.01 (0–14.10)	
60+	27	3.71 (2.27–4.15)	26.85 (9.24–44.46)	68.06 (49.83–86.29)	5.09 (0–12.21)		66.90 (48.45–85.35)	28.05 (10.76–45.34)	5.05 (0–14.67)	
Gender										
Woman/girl	197	25.35 (22.021–28.68)	41.32 (33.67–48.97)	48.95 (41.21–56.70)	9.73 (4.64–14.81)	0.6272	56.71 (49.13–64.29)	32.86 (25.71–40.02)	10.43 (5.52–15.34)	0.4621
Man/boy	186	24.19 (20.87–27.51)	38.18 (30.57–45.79)	52.99 (45.04–60.94)	8.83 (4.04–13.62)		54.52 (46.73–62.31)	39.45 (31.80–47.10)	6.03 (2.59–9.47)	
Indigenous or other cultural gender identity	17	2.22 (1.13–3.32)	52.29 (27.84–78.74)	38.27 (14.03–62.50)	8.44 (0–24.17)		54.09 (29.39–78.79)	35.21 (11.52–58.91)	10.70 (0–24.92)	
Nonbinary, genderqueer	379	48.66 (44.50–52.11)	44.00 (38.40–49.61)	50.23 (44.57–55.89)	5.77 (3.25–8.29)		61.26 (55.95–66.57)	30.84 (25.85–35.82)	7.90 (4.75–11.06)	
Sex assigned at birth						0.5673				0.4620
Female	517	66.22 (62.57–69.86)	42.11 (37.38–46.84)	50.97 (46.15–55.78)	6.92 (4.56–9.29)		58.83 (54.25–63.40)	34.11 (29.70–38.52)	7.06 (4.73–9.40)	
Male	256	33.78 (30.14–37.43)	42.20 (35.47–48.93)	48.48 (41.66–55.31)	9.32 (4.85–13.79)		56.73 (50.06–63.40)	33.34 (27.08–39.61)	9.92 (5.50–14.34)	
Racialized as a person of Color						0.0033^**^				0.5346
Yes	102	13.68 (11.01–16.35)	59.32 (48.66–70.00)	35.74 (25.38–46.10)	4.94 (0.16–9.72)		63.11 (53.03–73.19)	30.69 (20.98–40.39)	6.20 (1.64–10.77)	
No	676	86.32 (83.65–88.99)	39.39 (35.32–43.48)	52.50 (48.30–56.69)	8.10 (5.71–10.50)		57.44 (53.38–61.49)	34.10 (30.24–37.96)	8.47 (6.07–10.86)	
Indigenous						0.0254^*^				0.3914
Yes	52	7.99 (5.74–10.23)	52.99 (38.05–67.93)	31.97 (18.25–45.69)	15.04 (3.34–26.73)		66.82 (53.44–80.21)	27.67 (14.87–40.48)	5.50 (0.08–10.93)	
No	725	92.01 (89.77–94.00)	41.18 (37.19–45.16)	51.75 (47.69–55.81)	7.07 (4.95–9.20)		57.89 (53.68–0.25)	34.00 (30.27–37.74)	8.41 (6.10–10.72)	
Born outside of Canada						0.6118				0.5922
Yes	109	13.09 (10.61–15.57)	43.69 (33.43–53.94)	51.17 (40.86–61.49)	5.14 (0.58–9.69)		57.20 (47.24–67.16)	36.92 (27.19–46.65)	5.88 (1.54–10.22)	
No	666	86.91 (84.43–89.39)	41.67 (37.51–45.84)	50.20 (45.96–54.45)	8.13 (5.71–10.54)		58.53 (54.46–62.60)	33.14 (29.28–37.01)	8.33 (5.95–10.71)	
Below LIM threshold at prepandemic baseline^a^						<0.0001^**^				0.0893
Yes	328	44.22 (40.35–48.09)	53.24 (47.11–59.38)	38.30 (32.25–44.34)	8.46 (5.27–11.65)		63.20 (57.48–68.91)	29.42 (24.06–34.78)	7.39 (4.14–10.64)	
No	423	55.78 (51.91–59.65)	33.52 (28.59–38.45)	58.83 (53.63–64.03)	7.65 (4.50–10.80)		54.63 (49.50–59.76)	37.39 (32.42–42.36)	7.98 (5.17–10.80)	
Household income at follow-up						<0.0001^**^				0.0207^*^
No income	125	16.73 (13.86–19.61)	54.41 (44.74–64.08)	38.02 (28.62–47.42)	7.57 (2.01–13.13)		58.95 (49.64–68.25)	36.01 (26.90–45.11)	5.05 (0.92–9.18)	
<14,999	216	28.28 (24.81–31.75)	48.76 (41.18–56.34)	38.96 (31.43–46.49)	12.28 (7.01–17.55)		61.80 (54.72–68.89)	32.92 (26.02–39.82)	5.28 (2.26–8.29)	
15–29,999	160	21.37 (18.20–24.53)	49.56 (41.06–58.06)	46.06 (37.56–54.56)	4.38 (1.30–7.46)		57.34 (49.03–65.66)	29.06 (21.53–36.58)	13.60 (7.54–19.66)	
30–49,999	123	16.25 (13.39–19.11)	31.05 (21.93–40.18)	62.40 (52.78–72.02)	6.55 (1.16–11.93)		63.10 (53.91–72.29)	31.57 (22.70–40.44)	5.33 (1.40–9.27)	
50–79,999	93	10.72 (8.50–12.93)	19.17 (9.93–28.40)	73.93 (63.85–84.02)	6.90 (1.26–12.54)		41.22 (30.67–51.77)	49.29 (38.48–60.10)	9.49 (1.64–17.35)	
80,000+	53	6.65 (4.78–8.53)	26.26 (13.49–39.03)	70.72 (57.59–83.85)	3.02 (0–7.33)		62.26 (47.90–76.62)	28.30 (14.94–41.66)	9.44 (0.46–18.43)	
Education						0.0178^*^				0.5744
<High school	25	5.57 (3.39–7.5)	60.49 (39.39–81.59)	33.77 (13.54–54.00)	5.74 (0–16.65)		57.53 (37.61–77.44)	26.78 (9.21–44.35)	15.69 (1.28–30.10)	
High school diploma	64	11.16 (8.49–13.83)	48.01 (34.56–61.46)	50.80 (37.35–64.26)	1.19 (0–3.51)		64.99 (52.68–77.31)	29.50 (17.61–41.40)	5.51 (0.10–10.91)	
Some college or university	218	27.61 (24.26–30.97)	45.43 (38.28–52.59)	43.28 (36.06–50.50)	11.29 (6.55–16.04)		58.44 (51.44–65.44)	35.53 (28.76–42.30)	6.03 (2.41–9.64)	
College or university degree	343	42.14 (38.41–45.87)	40.35 (34.66–46.037)	52.34 (46.48–58.19)	7.31 (4.14–10.48)		55.93 (50.28–61.59)	34.27 (28.91–39.63)	9.80 (6.28–13.32)	
Grad/professional degree	130	13.51 (11.13–15.89)	28.65 (20.16–37.13)	64.18 (54.91–73.45)	7.17 (1.17–13.18)		59.97 (50.72–69.22)	33.67 (24.70–42)	6.35 (1.60–11.10)	

Composite financial change variable: If participants selected negative major/moderate impact on both items or negative major/moderate impact on one item and neutral or positive minor impact on another, they were categorized as Negative Change (0). If participants selected positive major/moderate impact on both items or positive major/moderate impact on one item and neutral or negative minor impact on another, they were categorized as Positive Change (2). If participants selected minor impact or neutral on both items, they were categorized as having experienced No Change/Impact (1). If participants selected negative major/moderate impact on one item and major/moderate positive impact on another, they were coded as missing. If participants selected minor or no impact on one item and did not respond to the other, they were coded as missing.

^a^ Calculated using Statistics Canada 2019 low-income measure, which adjusts household income by number of people supported by income.

Categorical variables were analyzed as nominal variables using SAS Class statement.

^*^ Significant at *p* < 0.05.

^**^ Significant at *p* < 0.01.

CI, confidence interval; LIM, low-income measure.

#### Survey weights and descriptive analyses

We weighted all analyses using Stata ipfweight, which performs stepwise adjustment to achieve known population margins. Our weights represented the full sample, including the short version-only respondents to the 2019 survey, to ensure that results were representative of the full study sample. Subsequent analyses were performed using survey procedures in SAS 9.4. We estimated weighted frequencies and used *t*-tests to assess changes in depression and anxiety from prepandemic baseline to pandemic follow-up, using nonimputed data.

We produced *χ*^2^ tests of independence to examine the associations between our outcomes (financial change and change in access to TNB peer gatherings) and age category, gender, sex assigned at birth, PoC (yes/no), Indigeneity (yes/no), being born outside of Canada (yes/no), falling below the Statistics Canada low-income threshold at baseline (Yes/No), household income at pandemic follow-up, and highest education at baseline.

#### Multivariable linear regression

We fit four multivariable regression models to examine the relationship between financial change and mental health (depression and anxiety symptoms) and then change in access to TNB peer gatherings and mental health. The *class* statement of SAS was used to classify categorical variables (e.g., gender) in the analysis as nominal. We weighted regression analyses after imputation. *R^2^* and *adjusted R^2^* were obtained by averaging output from the 20 imputations. Before analysis, we checked regression diagnostics and determined that 2019 income-to-needs ratio and prepandemic social support were skewed. A sensitivity analysis determined that the pattern of results with or without transformation were the same. We thus present the analyses without transformation.

We additionally examined an alternative coding approach for our financial change composite variable, in which we respectively pooled participants who reported negative impact to any degree (major/moderate/minor) and those who reported positive impact to any degree (major/moderate/minor) on both items, rather than pooling minor impact with neutral impact. A sensitivity analysis determined no notable difference in results.

#### Confounders

Confounders were selected using directed acyclic graphs created in DAGitty.net.^33,34^ Previous research and community knowledge were used to select variables that impact mental health, and factors that influence exposure variables. In models containing financial change as the exposure, confounders (measured at baseline) were age, gender, immigration history (yes/no), employment, education, income-to-needs ratio, Indigeneity (yes/no), province, and racial background. In models with change in access to TNB gatherings as the exposure, confounders were sex assigned at birth, province at baseline, racial background, gender, immigration history, baseline social support, and living with an unsupportive household member during the COVID-19 pandemic (yes/no).

## Results

### Sample characteristics and descriptive statistics

The mean age was 31.84 years (standard deviation = 11.02, range = 18–74, 95% confidence interval [CI] = 32.12–33.84). Other sociodemographic details and comparisons in change in finances and access to TNB peer gatherings across groups can be found in Table 1. Around 9.17% and 20.27% of our sample reported that COVID-19 had a major (95% CI = 6.88–11.46) or moderate (95% CI = 17.22–23.33) negative impact on ability to meet financial obligations or essential needs, respectively (Table 2). Around 17.6% and 16.4% of the sample reported that COVID-19 caused major (95% CI = 14.68–20.54) or moderate (95% CI = 13.54–19.18) negative impact on personal income, respectively. Around 58.3% (95% CI = 54.52–62.03) of participants reported loss in access to TNB peer gatherings since March 12, 2020 (Table 3).

**Table 2.** Weighted Proportions of Responses to Questions Regarding Impact of the COVID-19 Pandemic on Finances

Ranking	“Which of the following best describes the impact of COVID-19 on your ability to meet financial obligations or essential needs, such as rent or mortgage payments, utilities and groceries?”			“How much of an impact did the COVID-19 pandemic have on your personal income from employment?”		
	*n*	%	95% CI	*n*	%	95% CI
Major negative	66	9.17	6.88–11.46	133	17.61	14.68–20.54
Moderate negative	157	20.27	17.22–23.33	126	16.36	13.54–19.18
Minor negative	183	24.05	20.76–27.35	119	15.95	13.10–18.79
No change	233	30.08	26.57–33.58	287	37.50	33.77–41.24
Minor positive	58	7.92	5.75–10.09	55	7.38	5.32–9.54
Moderate positive	42	6.00	4.06–7.93	25	3.41	2.02–4.80
Major positive	19	2.51	1.27–3.74	12	1.80	0.61–2.99

**Table 3.** Weighted Proportions of Responses to the Question, “Since March 12, 2020, Has Your Access to Trans and Nonbinary Peer or Friend Gatherings (Online or In Person)…”

Ranking	n	%	95% CI
Decreased	459	58.27	54.52–62.03
Stayed the same	262	33.58	30.00–37.17
Increased	57	8.14	5.98–10.30

We found statistically significant differences in change in finances across racialization categories [*χ^2^*(2) = 13.75, *p* = 0.0033], wherein PoC reported the highest proportion of negative change (59.3%) and the lowest proportion in positive change (4.9%). Similarly, we found an association between financial change and Indigenous identity [*χ^2^*(2) = 10.39, *p* = 0.0254], with Indigenous participants reporting higher proportions in both negative (53.0%) and positive changes (15.04%) compared with non-Indigenous participants. There was a significant association between change in finances and income at prepandemic baseline [*χ^2^*(2) = 30.90, *p* < 0.0001] and pandemic follow-up [*χ^2^*(10) = 60.25, *p* < 0.0001], as well as with level of education at baseline [*χ^2^*(8) = 25.27, *p* = 0.0178]. Finally, we found a significant relationship between change in access to TNB peer gatherings and income during the pandemic [*χ^2^*(10) = 24.71, *p* = 0.0207; Table 1].

The majority (67.3%) of participants were above the CESD cutoff for depression symptoms at both time points. A paired-samples *t*-test revealed that mean depression scores increased significantly from prepandemic baseline (mean [*M*] = 14.47, 95% CI = 13.93–15.01) to pandemic follow-up (*M* = 16.50, 95% CI = 15.98–17.01), *t*(730) = 9.04, *p* < 0.0001. Around 17.4% of the sample exhibited a clinically meaningful increase in CESD scores, from below the cutoff (10) at baseline to above the cutoff at pandemic follow-up.

The majority (70.1%) of the sample was above the OASIS cutoff for anxiety symptoms at both time points. Around 9.5% of the sample exhibited a clinically meaningful increase in OASIS scores, from below the cutoff (8) at prepandemic baseline to above the cutoff at follow-up. A paired-samples *t*-test revealed that mean anxiety scores increased significantly from pre-pandemic baseline (*M* = 10.13, 95% CI = 9.80–10.46) to pandemic follow-up (*M* = 10.35, 95% CI = 10.04–10.66), *t*(730) = 2.29, *p* = 0.022.

### Multivariable regression analysis

#### Effects of change in finances

**FIG. 1.**
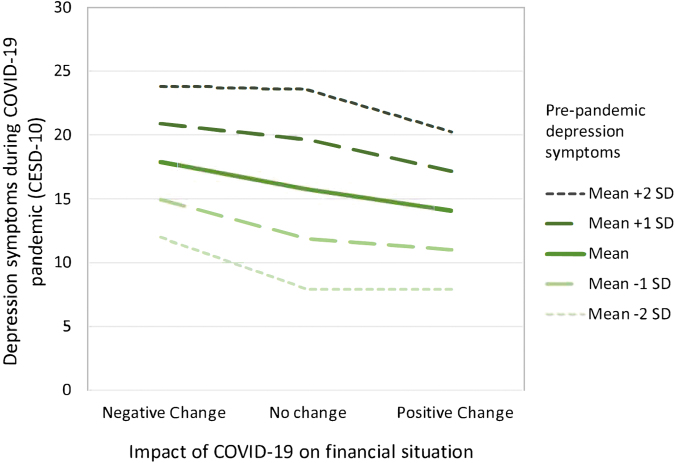
Estimating pandemic depression symptoms based on financial change for five levels of prepandemic depression scores. CESD-10, 10-item Center for Epidemiologic Studies Depression Scale; SD, standard deviation.

There was a statistically significant interaction between no change in finances and prepandemic depression on pandemic depression (*β* = 0.12, *p* = 0.0418; Table 4). Those with higher prepandemic depression had higher pandemic depression overall. However, those with a negative financial change experienced more depressive symptoms during the pandemic than did their peers at a similar level of prepandemic depression who experienced positive financial change. For those with no prepandemic depressive symptoms, having no financial change or a positive financial change were associated with a CESD-10 score 4.09 and 4.23 points lower, respectively (*p* < 0.0001 and *p* = 0.0385), when compared with those who had a negative financial change. This effect varied across different levels of prepandemic depression (See Fig. 1 for a graph estimating pandemic depression based on financial change for five levels of prepandemic depression.). This model explained 38% of variance in depression.

**Table 4.** Predicting Mental Health During the COVID-19 Pandemic with Perceived Financial Change and Prepandemic Mental Health (*n* = 780)

	****Outcome****: Depression symptoms during the pandemic (CESD-10)			****Outcome****: Anxiety symptoms during the pandemic (OASIS)		
	Coefficient	95% CI	*p*	Coefficient	95% CI	*p*
Intercept	14.10	10.58–17.61	<0.0001	5.52	3.30–7.74	<0.0001
Financial change						
Negative	Reference			Reference		
No change	−4.09	−6.10 to −2.08	<0.0001^**^	1.06	−2.62 to 0.50	0.1844
Positive	−4.23	−8.24 to −0.22	0.0385^*^	1.04	−1.75 to 3.84	0.4636
Prepandemic CESD-10	0.43	0.34–0.53	<0.0001^**^	—	—	—
Prepandemic OASIS	—	—	—	0.59	0.49–0.69	<0.0001^**^
Prepandemic CESD × financial change				—	—	—
CESD-10 × negative	Reference					
CESD-10 × no change	0.12	0.002–0.26	0.0418^*^			
CESD-10 × positive	0.02	−0.24 to 0.27	0.8948			
Prepandemic OASIS × financial change	—	—	—			
OASIS × negative				Reference		
OASIS × no change				0.05	−0.07 to 0.18	0.4992
OASIS × positive				−0.21	−0.51 to 0.08	0.1500
*R^2^*	0.40			0.044		
Adjusted *R^2^*	0.38			0.041		

Financial change is coded as: Negative = 0, No change = 1, Positive change = 2.

All models control for age, 2019 income-to-needs ratio, gender (man/boy = 1, woman/girl = 2, Indigenous or other cultural gender identity = 3, nonbinary, genderqueer, agender, or similar = 4), immigration status (1 = yes, 0 = no), racialization as a person of Color (1 = yes, 0 = no), Indigeneity (1 = yes, 0 = no), baseline employment (1 = permanent full-time, 2 = employed but not permanent full-time, 3 = not employed or on leave, 4 = not employed and student or retired), province (1 = British Columbia, 2 = Alberta, 3 = Saskatchewan, 4 = Manitoba, 5 = Ontario, 6 = Quebec, 7 = New Brunswick, 8 = Prince Edward Island, 9 = Nova Scotia, 10 = Newfoundland and Labrador, 11 = Northwest Territories, Nunavut, Yukon), baseline education (1 = Less than high school, 2 = high school diploma or equivalent certificate, 3 = Some college or university, 4 = College, university, or equivalent degree, 5 = Graduate or professional degree).

Categorical variables were analyzed as nominal variables using SAS Class statement.

^*^ Significant at *p* < 0.05.

^**^ Significant at *p* < 0.01.

CESD-10, 10-item Center for Epidemiologic Studies Depression Scale; OASIS, Overall Anxiety Severity and Impairment Scale.

We found no similar interaction effect between prepandemic anxiety and (positive, *p* = 0.1500 or no, *p* = 0.4992) financial change on pandemic anxiety. Change in finances did not significantly contribute to predicting follow-up anxiety, but higher prepandemic anxiety was associated with higher pandemic anxiety (*β* = 0.59, *p* < 0.0001).

#### Effects of change in access to TNB peer gathering

**FIG. 2.**
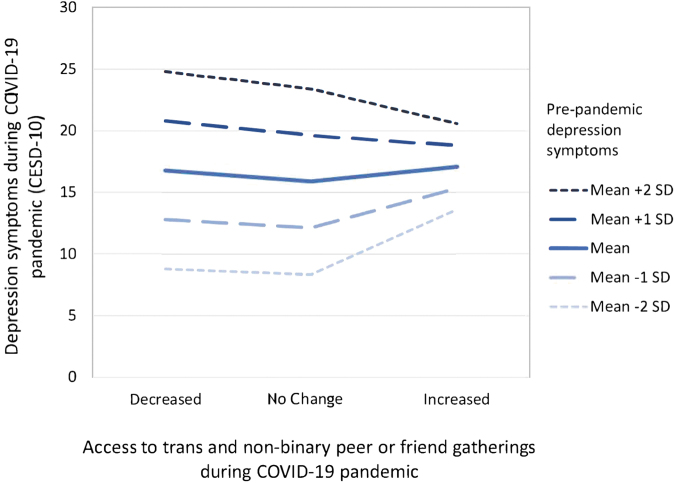
Estimating pandemic depression symptoms based on change in access to trans and nonbinary peer gatherings for five levels of prepandemic depression scores.

There was a significant interaction between increase in access to TNB peer gatherings and prepandemic depression on pandemic depression (*β* = −0.33, *p* = 0.0004; Fig. 2 and Table 5). Those with higher prepandemic depression had higher pandemic depression overall. Participants with greater prepandemic depression and increased access to TNB peer gatherings showed lower depressive symptoms compared with those with decreased access to TNB peer gatherings. However, among participants with low prepandemic depression, increased access to TNB peer gatherings appeared to be associated with more depression symptoms during the pandemic compared with decreased access to TNB peer gatherings (See Fig. 2 for a graph estimating pandemic depression based on change in access to TNB peer gatherings for five levels of prepandemic depression.). This model explained 28% of variance in depression.

**Table 5.** Predicting Mental Health During the COVID-19 Pandemic with Perceived Change in Access to Trans and Nonbinary Peer Gatherings (*n* = 780)

	***Outcome***: Depression symptoms during the pandemic (CESD-10)			***Outcome***: Anxiety symptoms during the pandemic (OASIS)		
	Coefficient	95% CI	*p*	Coefficient	95% CI	*p*
Intercept	9.41	6.32–12.51	<0.0001	14.93	11.68–18.18	<0.0001
Change in access to TNB peer gatherings						
Decrease	Reference			Reference		
No change	−0.41	−2.44 to 1.63	0.7001	−1.29	−3.84 to 1.84	0.0956
Increase	5.03	2.05–8.01	0.0019^**^	0.12	−2.73 to 6.20	0.9380
Prepandemic CESD-10	0.58	0.50–0.67	<0.0001^**^	—	—	—
Prepandemic OASIS	—			0.59	0.51–0.85	<0.0001^**^
Prepandemic CESD-10 × change in gathering access						
CESD-10 × decrease	Reference			Reference		
CESD-10 × no change	−0.034	−0.16 to 0.09	0.5368			
CESD-10 × increase	−0.33	−0.51 to −0.15	0.0004^**^			
Prepandemic OASIS × change in gathering access	—	—	—			
OASIS × decrease				Reference		
OASIS × no change				0.09	−0.05 to 0.22	0.2148
OASIS × increase				−0.08	−0.19 to 0.35	0.5713
*R^2^*	0.30			0.52		
Adjusted *R^2^*	0.28			0.50		

All models control for gender (man/boy = 1, woman/girl = 2, Indigenous or other cultural gender identity = 3, nonbinary /genderqueer = 4), immigration status (1 = yes, 0 = no), racialization as a person of Color (1 = yes, 0 = no), province (1 = British Columbia, 2 = Alberta, 3 = Saskatchewan, 4 = Manitoba, 5 = Ontario, 6 = Quebec, 7 = New Brunswick, 8 = Prince Edward Island, 9 = Nova Scotia, 10 = Newfoundland and Labrador, 11 = Northwest Territories, Nunavut, Yukon), baseline social support (Medical Outcomes Social Support Scale), sex assigned at birth (1 = male, 2 = female), report of living with unsupportive household member (1 = yes, 2 = no).

Categorical variables were analyzed as nominal variables using SAS Class statement.

^*^ Significant at *p* < 0.05.

^**^ Significant at *p* < 0.01.

TNB, trans and nonbinary.

Similar to findings in our examination of financial change, change in access to TNB peer gatherings did not significantly contribute to the model with pandemic anxiety as an outcome, but prepandemic anxiety was associated (*β* = 0.59, *p* < 0.0001).

## Discussion

In the present study, we examined associations between changes in finances and access to TNB peer gatherings and mental health during the COVID-19 pandemic. Approximately half of our sample reported that COVID-19 had a negative impact on finances. The majority reported a loss in access to TNB peer gatherings (online and in person). Although the present study examined only symptomology and was not a clinical study, we found that both depression symptoms and anxiety severity/impairment increased significantly from prepandemic baseline to pandemic follow-up, and that most participants were above recommended cutoffs for both depression and anxiety at both time points. When observing pandemic depression symptoms we found that they were affected by changes in finances and access to TNB peer gatherings, and that these impacts varied depending on prepandemic depression. In contrast, these factors did not predict pandemic anxiety, which appeared to be a function of prepandemic anxiety.

Our findings substantiate previous research showing that income loss during COVID-19 was linked with reduced mental health among TNB people^19^ and that prepandemic mental health predicted pandemic mental health.^35–37^ Our results advance this knowledge, revealing an interaction between prepandemic depression and financial change. For individuals low in depression symptoms before the pandemic, financial stability and positive change were protective, predicting fewer depressive symptoms compared with that predicted by a negative change. Meanwhile, for individuals who entered the COVID-19 pandemic with high depression symptoms, only positive financial change predicted reduced depressive symptoms; financial loss and stability predicted similarly high scores in depression.

Past research has noted complex associations between mental health and finances. For example, research on employment security and mental health of low-income, urban women showed that employment security was only protective of mental health if the women had access to childcare.^38^ In other words, financial stability does not directly improve mental health, and the relationship between financial status and mental health is complicated by other factors (e.g., tangible support).

We found that the relationship between change in access to TNB peer gatherings and pandemic depression varied based on prepandemic depression. Participants low in prepandemic depression symptoms who experienced an increase in access to TNB peer gatherings reported greater pandemic depression symptoms than those with low prepandemic depression and a decrease or no change in access to TNB peer gatherings. It is possible that participants with low prepandemic depression pursued more community engagement during the COVID-19 pandemic because they were experiencing increased depression symptoms; in this case, increased depression may have led to increased access to TNB peer gatherings. Meanwhile, individuals who entered the pandemic with greater depression symptoms and reported an increase in access to TNB peer gatherings experienced the reduced depression symptoms that might be expected.

In line with these findings, research with a nationally representative sample in the United States has shown that building social trust with surrounding community was protective for people with major depression at prepandemic baseline, but not for those without depression at prepandemic baseline.^39^ Results from the same study showed that, while trust was protective for people with prepandemic depression, community participation was not—suggesting that merely having access to community may not be protective and highlighting the importance of separating social capital into different dimensions when examining its relationship with mental health.^39^ For example, it would be helpful to better understand the impact of cognitive (e.g., belief in the likelihood of receiving support), structural (e.g., participating in local associations), and bonding versus bridging dimensions of social capital (e.g., closeness with similar others vs. relationships that transcend identity categories)^39^ on TNB mental health.

Neither of our predictors were significantly associated with pandemic anxiety, and prepandemic anxiety was the primary predictor of pandemic anxiety, which is consistent with earlier research.^24^ Our results suggest that anxiety persists, regardless of change in finances or access to TNB peer gatherings, whereas pandemic depression may be assuaged or enhanced. This is in line with other COVID-19-related research which showed that anxiety, but not depression, increased during the COVID-19 pandemic.^40^ Anxiety tends to persist more than other forms of distress, and is less likely to be moderated by other factors (e.g., social support).^41^ In a study with patients with multiple sclerosis, distress decreased over time, but high levels of anxiety did not change—a finding that the authors attributed to uncertainty about the future.^42^ Uncertainty was a major contributor to anxiety during COVID-19,^43^ and may be one reason that anxiety persisted in our sample, regardless of change in finances or access to TNB peer gatherings.

Participants who were PoC, Indigenous, or low-income were more likely to report negative financial change—which is in line with results from previous reports conducted in Canada and the United States on wage and job loss during the COVID-19 pandemic.^44,45^ Previous research has demonstrated that TNB PoC—who encounter compounded and intersecting systems of oppression—are more likely than their White counterparts to live below the poverty line and be unemployed.^5^ Relatedly, COVID-19 rates were higher among PoC and Indigenous peoples compared to White and non-Indigenous counterparts (for reasons that include overcrowded living conditions, unsafe and/or front-line work, barriers to equitable health care),^46^ which may also impact finances. These effects of racism and transphobia, and related intergenerational and economic oppressions, may have positioned TNB individuals who are low-income, Indigenous, and/or PoC to be particularly vulnerable to the economic loss and instability caused by COVID-19.

The majority of our sample reported depression and anxiety symptom scores that were above established clinical cutoff points both during and before the pandemic. Previous research has demonstrated that negative mental health outcomes among TNB individuals are linked to systemic oppressions, being unable to actualize one's gender, lack of support, absence of protective workplace policies, and difficulty accessing TNB-specific health care services.^47^ The COVID-19 pandemic did not originate these issues, but made them starker, for example, by pushing individuals to lockdown with unsupportive families of origin and limiting access to TNB care.^48,49^

In response to the COVID-19 pandemic, the Canadian government made funds available to support mental health needs that would otherwise have been met via support networks that were closed during the pandemic.^50^ Funding was also made available to community groups, with the objective of increasing COVID-19 vaccine uptake.^50^ However, our findings suggest that these generic and temporary programs may be an inadequate solution to preexisting and increased mental health concerns among TNB individuals during the pandemic.

### Limitations

The present study had many strengths, including a longitudinal design and a diverse sample. It is worth noting that, while there are common mental health factors shared by the TNB population (e.g., freedom to express gender), other variables often differ across groups (e.g., discrimination, resilience, and health outcomes experienced by binary vs. nonbinary trans people),^51,52^ which presents a limitation when pooling these populations. Approximately one quarter of our sample reported being under the age of 25 and a similar proportion reported completing some college/university; thus, it is possible that these individuals were financially dependent on a parent or guardian and that they, similar to individuals under 18, may not have been directly or as dramatically impacted by financial changes during the pandemic.

Our predictors examined perceived change during the COVID-19 pandemic (e.g., in ability to afford needs, access to TNB peer gatherings), rather than measuring change in these variables before and during the pandemic, which may have negatively impacted the accuracy of our investigation. For example, some individuals may have reevaluated and reduced their expenses during the pandemic (e.g., by moving into less expensive housing), resulting in a report of income loss, but little impact on ability to afford needs. The 2019 income-to-needs ratio was calculated using income categories that had varying intervals, which could only result in an approximation. Furthermore, incomes under $10,000 and $150,000 or more were considered $9999 and $150,000, respectively; yet these integers may not have reflected true income due to the breadth of the categories.

In addition, our measure of access to TNB peer gatherings did not specify the nature of this access; however, the nature of social engagement among marginalized groups may determine different mental health outcomes.^53^ Access to TNB peer gatherings may also have been in the form of social media “lurking,” which may have a negative relationship with perceived social support and be related to worsened mental health.^54^ This limits the interpretability of our findings.

TPC was conceptualized and executed with an intersectional framework (see Crenshaw^55^), employing a community-based approach with priority populations and an overarching emphasis on the effects of systemic oppression on health inequity.^56^ Prior research has suggested that TNB individuals facing multiple intersecting systems of oppression (e.g., racism, ableism, classism) may be at greater risk of health inequity^57,58^ and that the potential benefits of community resources may vary across groups.^59^ Due to limited sample size, we did not conduct an intersectional analysis in the present report, which limited our ability to investigate potential variations across subgroups and obtain deeper understanding into the experiences of the diverse TNB population. We additionally were unable to include participants who did not complete the COVID-19 follow-up survey and it is possible that their unavailability was related to distress or financial strain.

## Conclusion

The immediate and long-term consequences of the COVID-19 pandemic must be considered within the context of existing health inequity and structural mechanisms that already disadvantaged the TNB population. In the present study, the relationship between pandemic depression and changes in finances and access to TNB peer gatherings varied, depending on prepandemic depression. Meanwhile, anxiety persisted and increased, regardless of change in finances or access to TNB peer gatherings. Before and during COVID-19, depression and anxiety metrics surpassed clinical cutoffs, which evidences the consequences of systemic oppressions that were then amplified during the pandemic. These findings suggest that minor or temporary financial assistance is likely an oversimplified solution to increased mental health concerns during the COVID-19 pandemic.

TNB individuals may benefit from multifaceted approaches to mental health programs and services that address multiple dimensions of need (e.g., financial support and meaningful community engagement) and respond to economic oppression and health inequity. The TNB population may benefit from more targeted resources and tools that address specific needs and dismantle barriers, such as workplace, housing, health care, and community policies that protect the safety and security of TNB individuals. Such interventions may include financial support to obtain independent housing, virtual TNB community spaces, and free or affordable professionally moderated groups that offer community connection and mental health support. Continued access to hormone therapy and other health care via telemedicine would be impactful at any time—but especially during a pandemic.

## Data Availability Statement

The 2019 TPC data cannot be deposited in a data repository due to conditions in the letter of information and consent for participants that specified data would only be seen by members of the large national team's Data Analysis Working Group. These conditions were necessary in the context of stigmatization and distrust of researchers. A 2020 COVID-19 data subset is available through an interactive dashboard at https://transpulsecanada.ca/covid/
